# Supplementary material for: ImmunoMatch learns and predicts cognate pairing of heavy and light immunoglobulin chains
Source: Nat Methods. 2025 Nov 18;23(1):106–17. doi: 10.1038/s41592-025-02913-x (PMC12791012; doi:10.1038/s41592-025-02913-x)
Supplement: Supplementary file 1 — Supplementary Tables 1–4, Notes 1–2, Figs. 1–7 and Methods. [file 41592_2025_2913_MOESM1_ESM.pdf]

nature methods

Supplementary information

https://doi.org/10.1038/s41592-025-02913-x

ImmunoMatch learns and predicts cognate pairing of heavy and light immunoglobulin chains

In the format provided by the authors and unedited

Supplementary Information

Contents

Supplementary Tables **2**

Supplementary Note 1: Impact of training datasets on ImmunoMatch performance **3**

Problem formulation in ImmunoMatch, training data and impact on metric choices . . . 3

Expanding the training data of ImmunoMatch 4

Incorporating different studies in the training data 7

Supplementary Note 2: Comparison of different single cell sequencing techniques using ImmunoMatch **9**

Supplementary Methods **11**

Predicting H-L pairing preference using CDR3 sequences 11

Predicting H-L pairing preference using V region sequences 12

Supplementary Tables

Table S1: Data collection for paired antibody sequences

Publication	Data source	Number of paired sequences	Number of pairs after clustering and sampling
**Rajan et al. (1)**	Donor1, cell type not annotated	3,177,863	20,100
	Donor2, cell type not annotated	2,528,133	20,100
**DeKosky et al. (2)**	Donor1, antigen experienced	30,131	20,100
	Donor2, antigen experienced	69,120	20,100
**Jaffe et al. (3)**	Donor3, Naïve B cells	161,327	20,100
	Donor3, Memory B cells	44,097	20,100

Supplementary Note 1: Impact of training datasets on ImmunoMatch performance

In this supplementary note we explored how training data choices can contribute to the performance of ImmunoMatch, in terms of both the scale and the composition of the data.

Problem formulation in ImmunoMatch, training data and impact on metric choices

A major distinction of ImmunoMatch in comparison to standard binary classification tasks is that the negative labels were constituted by synthetic examples, generated via shuffling observed H-L pairs. This is an unavoidable choice we made, as H and L chains which could not form stable and functional pairs were naturally removed during B cell development. We carefully considered the consequence of this in relation to evaluating the performance of ImmunoMatch: the use of synthetic negatives implies that the model has never really seen the true diversity of negatives, and that the false positive rate (FPR) when applying ImmunoMatch on real data is likely underestimated. Consequently, the model may hallucinate positives where they don't exist, because it only learned to reject "pseudo" negatives. To mitigate this, a low FPR is important in determining whether the assembled training data is appropriate, and whether the final model is properly trained. Additionally, given it is likely that users will apply ImmunoMatch on large repertoires (to prioritise a small number of H-L pairs potentially for experimental investigation), a low FPR also ensures that H-L pairs that are taken forward for more detailed experimental characterisation are likely true pairs that could be subject to in-depth investigations, for applications such as subsequent engineering to improve stability and specificity.

The ImmunoMatch models presented in the text were trained with paired H-L data from n = 6 donors from three datasets. We made the explicit choice of including only healthy individuals in the training set to minimise potential bias diseases and immune conditions would pose on altering H-L pairing preferences. Although only healthy individuals were seen during training, we have shown in use cases presented in the main text that the ImmunoMatch models could also be applied on H-L sequences sampled from diseased states, for instance in different leukaemia and lymphoma as well as infiltrating B cells found in solid tumours. As single-cell sequencing approaches are increasingly adopted in the study of antibody immunity, and the scale of such datasets continuously expands, we expect more data will be available that could be useful in improving ImmunoMatch's performance. While we will continue to monitor for such datasets, we also need to consider whether incorporating new data will necessarily contribute to sufficient gain in predictive performance that is commensurate to the increase in data scale, for instance in whether the quality of such datasets will be beneficial to the classification task and provide improvement to metrics such as FPR that one should monitor in the context of the problem we formulated.

Expanding the training data of ImmunoMatch

We first investigated the incorporation of additional healthy donors to train ImmunoMatch. These additional data came from three other studies (4; 5; 6), not originally available when we trained the original ImmunoMatch models. This increased the number of donors for training from *n* = 6 to *n* = 11 (Table S2). However, we noted that some of these additional donors had very limited number of paired H-L sequences. We therefore included 20,100 pairs from each donor whenever possible (Table S2).

Table S2: Expanding training data for ImmunoMatch. New data were curated from: Phad et al. (4); James et al (5), and; Eccles et al. (6)

	**Original ImmunoMatch**	**ImmunoMatch with expanded data**
**Number of donors**	$n = 6$	$n = 11$
**Number of true H-L pairs considered**	120,600 (= 20,100 pairs per donor × 6)	164,527 (= 20,100 pairs per donor × 8 + small datasets [see below])
**Datasets with small number of true H-L pairs**	N/A	• James et al 2020 donor 417c (n = 2,687 pairs)<br>• James et al 2020 donor 390c (n = 300)<br>• Eccles et al 2020 donor d1 (n = 740)

Ideally, expanding the dataset should (a) improve the identification of true pairs (i.e. higher precision and recall); (b) decrease both the false negative and the false positive rates (since now exposing the model to more donors should make it more generalisable). We evaluated both models with three donors from the Jaffe et al. (3) dataset which we have used in the main text as an external test set (main text Figure 1g, "different donors"; note: the new model trained on the expanded data did not include these three donors). The results are shown in Figure S1 below. Whilst both models exhibit good recall (i.e. true H-L pairs being predicted correctly), surprisingly the model trained on the expanded dataset ("ImmunoMatch_extended" in Figure S1) generated more false positive (FP) predictions (top right corner in the confusion matrices).

There may be multiple factors which might contribute to the increase in FP, including differing distributions of cell types, data quality etc. In Figure S2a, we provide evaluation metrics for both models in this external test set. We observe an increase in accuracy, as well as a decrease in false negative rate with the ImmunoMatch_extended model, in comparison to the original. However, the false positive rate is higher in the "extended" model. As a negative control for comparison, we trained a reduced version of ImmunoMatch (ImmunoMatch_one-donor), where only antibodies from one donor (*n*=20,100) were utilized. ImmunoMatch_one-donor is unable to distinguish the paired H-L from unpaired, indicated by accuracy of 0.51 and AUC-ROC 0.52 (Figure S2a).

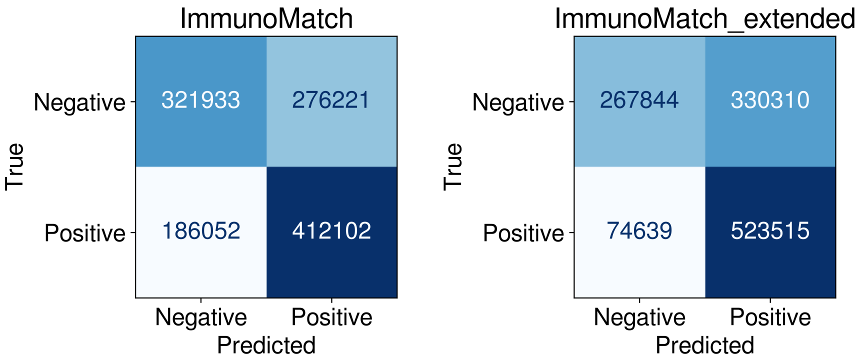

Figure S1: Confusion matrices from the prediction generated using the original ImmunoMatch (left) and the version of ImmunoMatch trained with an expanded training dataset (right, "ImmunoMatch_extended" from n = 11 donors). These results correspond to a test set of sequences from n = 3 donors from Jaffe et al. (3) (identical dataset as in main text Figure 1g, 'different donors'). These sequences were not included in deriving both models tested here. Numbers on the confusion matrices correspond to the number of test examples in each category.

As per standard practice in evaluating machine learning models we also computed equivalent metrics on test sequences withheld from each training dataset (Figure S2b). However, the performance on the withheld test set is not comparable across models because each of these models were trained on a different training data compilation. Another key concern is while withheld test sets are useful for assessing whether a model has learned meaningful features from the training data (i.e., to diagnose model underfitting), external test sets are critical for evaluating generalization and identifying potential overfitting. An overfitted model can perform well on withheld test set, but fail to generalise on external data. For example, ImmunoMatch_one-donor performs the best on all the matrices when tested on withheld test set (Figure S2b). However, the performance significantly drops and becomes the worst among these three models when evaluated on external test sequences, a clear sign of overfitting. This is problematic as external test sets better simulate real-world scenarios, i.e. when users apply ImmunoMatch to their own data. For these reasons, we compare these models based on their performance on the external test set.

We can see that the original ImmunoMatch (n = 6 donors) model is substantially better than the one-donor model, and its performance already approaches a plateau as evidenced by the ROC curve when tested on external test set. In rationalising the benefits and costs in expanding the training set, and considering the criteria we set at the beginning in selecting the training set, our main concerns are:

- Expanding the training set from 6 donors to 11 donors does not deduce the false positive rate.
- The tradeoff between expanding the training dataset and the consequent gain in performance is not ideal. Here for ImmunoMatch_extended, we have increased the size of the dataset by 36.4% (from 120,600 to 164,527 sequence pairs), with a gain in AUC-ROC by 0.04 on the external test set.

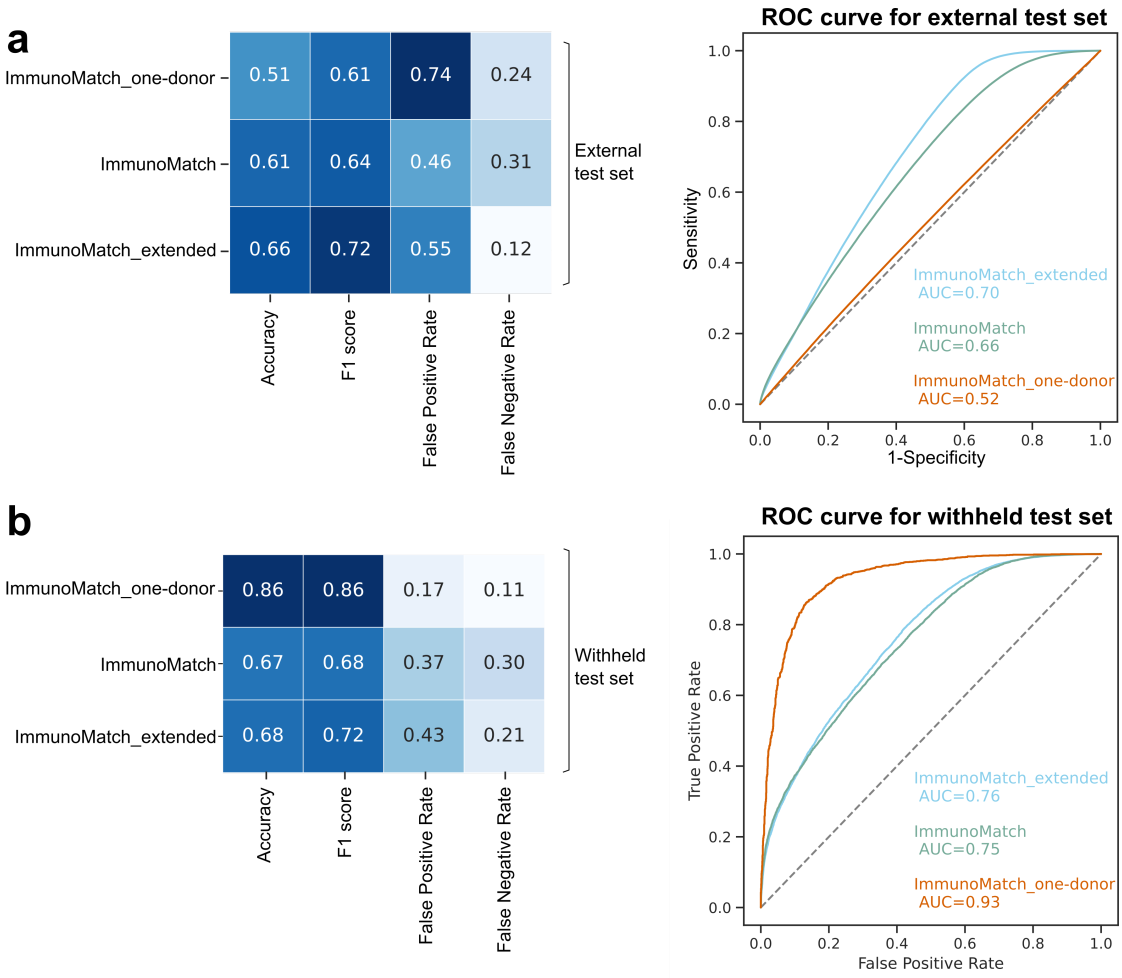

Figure S2: Evaluation metrics and Receiver Operating Characteristic (ROC) curves of the ImmunoMatch_one-donor, the original ImmunoMatch, and the ImmunoMatch_extended models (a) on external test data from the three withheld donors in Jaffe et al. (3). (b) on respective withheld test set on each model

Incorporating different studies in the training data

The quality of the training data obtained from different publications can vary due to different samples, experimental methods, etc. applied in each study. To investigate whether studies included in the current training set are informative in learning relevant signals indicative of H-L pairing, we trained models with sequences sampled from different combinations of studies and evaluated their performance. The original training set of ImmunoMatch includes sequences collected from three studies (3; 2; 1). Together with the newly added sequences from another three publications (4; 5; 6), there are sequences from six studies in total. To keep the number of studies consistent with the original ImmunoMatch, we sampled three out of six studies, leading to 20 unique combinations (Figure S3a). The sequences of each combination are split into training and withheld test sets, followed by the same protocol adopted to train the original ImmunoMatch, resulting in 20 trained models. Model 4 shares the same training set as the original ImmunoMatch.

As pointed out at the beginning of the supplementary note, false positive rate (FPR) should be the main concern when evaluating the impact of the dataset in training the model in this application. ImmunoMatch has the lowest FPR among the 20 models examined in the same external test set (Figure S3b). The FPR of ImmunoMatch when tested on the withheld test set is moderate among 20 models. We also provide analogous evaluation in the withheld sequences from each of the 20 models; however, as we detailed above, since the composition of the withheld test set varies between models in our case, this is not a fair comparison. The evaluation on the external test set aligns closer to the scenario in which the user applies ImmunoMatch to the real data they collected. Therefore, we believe that the current version of ImmunoMatch gives the best FPR among all the models tested.

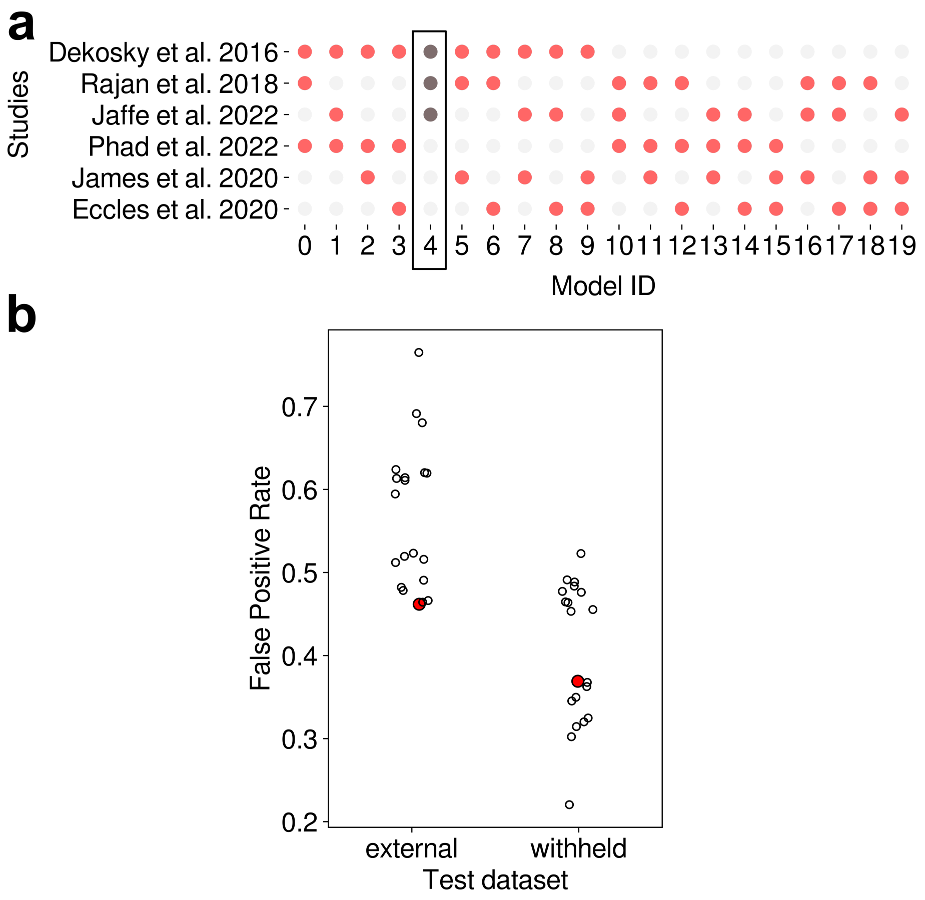

Figure S3: The performance of ImmunoMatch when training data is sampled from different studies. (a) Sampling datasets collected from 3 studies among 6 lead to 20 combinations. These combinations are inputted into the same protocol as the one used to train ImmunoMatch and resulted in 20 models. Model 4 shares the same training dataset as ImmunoMatch. (b) The evaluation of the false positive rate on both withheld test set and external test set. The performance of ImmunoMatch is highlighted in red.

In summary, considering all the criteria we applied in defining ideal training data for ImmunoMatch, we believe that the current version of ImmunoMatch used the selection of existing training data that optimise for this classification task, as evidenced by the minimisation of FPR on external test sequences. We will routinely monitor newly available datasets and review whether ImmunoMatch should be updated in view of new data.

Supplementary Note 2: Comparison of different single cell sequencing techniques using ImmunoMatch

In this Supplementary Note we explore whether ImmunoMatch can be used to compare different single-cell library generation protocols in their resolution of H-L pairing in the data. In standard single-cell datasets there are a small but significant proportion (5-10%) of cell barcodes mapped to more than 1 H and 1 L chain, i.e. these B cells can theoretically produce more than 1 unique BCR H-L pair. While some of these cases would correspond to technical artefacts (e.g. doublets being assigned the same cell barcode), they could reflect interesting biology (e.g. B cells expressing dual light chains potentially confer autoimmunity, see (7)). These observations are typically removed prior to further analysis, e.g. in (8). Even when such cases are not the cells of interest in the investigation, this nevertheless leads to smaller sample sizes that can be especially detrimental, e.g. when looking for rare B cell subtypes or antigen-specific B cells.

For a given cell barcode, if x H chains and y L chains are expressed, theoretically there can be at most xy H-L pairs associated with this cell barcode. Here we investigated whether ImmunoMatch can clarify these scenarios, by predicting different pairing scores for these theoretical pairs, and therefore allow us to resolve likely H-L pairs for these barcodes. To simplify interpretation, here we focus on comparing single-cell BCR pairs from healthy individuals obtained using different single-cell library preparation methods. Well-resolved single-cell data will have a clear peak of pairing score above 0.5 (ideally close to 1, depending on the proportion of naive and memory B cells, see main text **Figure 4a**), whilst data without such peak and the score distribution being muddled with spurious pairs will be typical of poorly-resolved single-cell data.

We compared single-cell BCR sequencing data generated using the following methods:

- SMART-seq2 (data from (9)): each single B cell is sorted into a well on a multi-well plate, and RNA-seq library preparation reactions are performed within individual wells.
- 10x Genomics (example dataset provided officially by the manufacturer[1]): individual cells are encased in lipid droplets, and cells and molecular barcodes tag transcripts within the droplet. Singlet droplets are obtained by controlling the rate of flowing through oil to mix with the cell suspension.
- Parse Biosciences (example data from healthy donors provided officially by the manufacturer[2]): cells are repeatedly split and harvested from multi-well plates tagged with barcodes; single cell resolution is achieved by combinatorially assembling the nucleotide indices into a unique sequence.

[1] https://www.10xgenomics.com/datasets/human-b-cells-from-a-healthy-donor-1-k-cells-2-standard-6-0-0

[2] https://www.parsebiosciences.com/datasets/bcr-sequencing-of-1-million-healthy-and-diseased-samples-in-a-single-experiment/

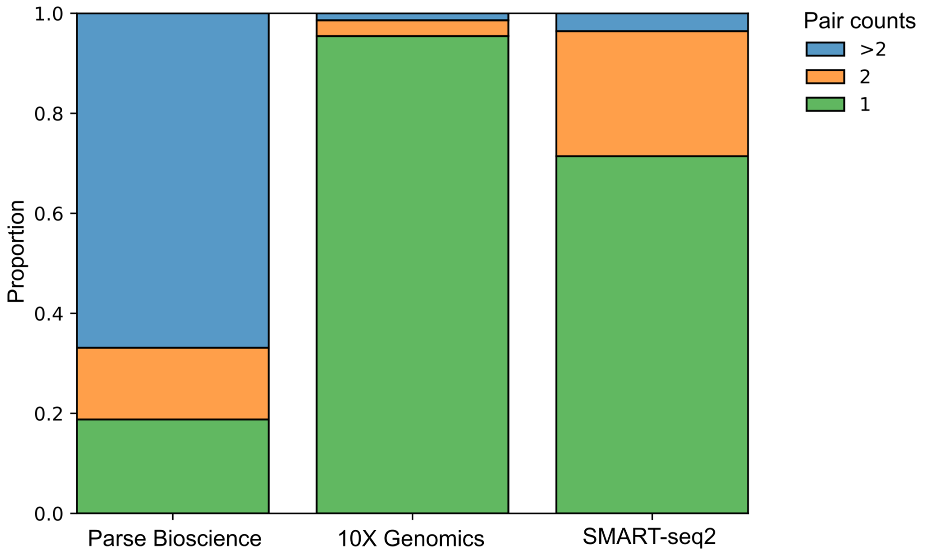

Figure S4: Enumeration of unique H-L pairs per cell barcode ("pair counts") in the Parse Bioscience, 10x Genomics and SMART-seq2 dataset of single-cell BCR repertoires analysed herein.

For each method we grouped the datasets into those with 1 unique H-L pair with a unique cell barcode (pair count = 1), 2 H-L pairs per barcode (pair count = 2) and those with >2 H-L pairs per barcode. We observed indeed that from the perspective of capturing single, unique H-L pair per cell barcode, these methods have different resolution (Figure **S4**).

We proceeded to score each H-L pair using ImmunoMatch-κ and ImmunoMatch-λ, given the light chain type in question. The results are shown in Table **S3**.

Table S3: ImmunoMatch analysis on paired BCR data generated using SMART-seq2, 10x Genomics and Parse Bioscience protocols.

	No. of. cell barcodes analysed	**% pairs with ImmunoMatch score $\geq$ 0.5**		
		Pair count = 1	Pair count = 2	Pair count >2
**SMART-seq2**	84	85.0%	85.7%	78.6%
**10x Genomics**	1,077	81.4%	75.0%	78.3%
**Parse Biosciences**	7,050	47.1%	40.0%	33.8%

The data align with the trends of reported doublet rates from 10x Genomics[3] and Parse[4]) (Figure **S4**). We can use the ImmunoMatch pairing score to resolve pairings in cases where pair count > 1, and also to identify promiscuous chains (i.e. similarly high pairing scores [> 0.5] when the same chain is paired with different partner chains as input to ImmunoMatch) to investigate underlying features in the immunoglobulin sequence and structure that explains this promiscuity.

[3]see information from 10x Genomics website for v2 and v3 chemistries

[4]https://support.parsebiosciences.com/hc/en-us/articles/360053107311-What-is-the-expected-doublet-rate

Supplementary Methods

Predicting H-L pairing preference using CDR3 sequences

One-hot encoding of the CDR3 sequences

One-hot encoding is a widely used technique to transform textual data into numerical vectors, enabling subsequent processing by machine learning models (10). Here, the CDR3 sequences were first one-hot encoded, with each amino acid represented by a vector of length 20, with nineteen `0`s and one `1` at the position of the amino acid in the sequence. For batch processing, sequences were padded with `0`s to achieve uniform lengths. Specifically, CDRH3 sequences were padded to 26 residues, and CDRL3 sequences to 14 residues. The padding strategy adopts the same way as the IMGT-established numbering scheme, where the gaps were inserted in the middle of the CDR3 sequences (11). After one-hot encoding, sequences were transformed to matrices of shape $(26, 20)$ for the H chain, $(14, 20)$ for L chain and inputted into the CNN model for training.

Convolutional neural network model architecture

The architecture of the CNN model is illustrated in Figure **S5**. Each pair of one-hot encoded CDRH3 and CDRL3 sequences was individually fed into three convolutional layers, with the first two layers followed by a max pooling layer. Convolutional layers aim to capture the local features of the input data, while max pooling layers aggregate these features by selecting the local maxima and reducing the data dimension. Parameters in convolutional layers were filters being 128, kernel size being 3, with rectified linear unit (ReLU) (12) as the activation function. The setup for the max pooling layer was stride being 3, pooling size being 3. The output of the final convolutional layer was then flattened and processed through four dense layers, with units 256, 256, 128 and 64 and RELu as the activation function. Each dense layer employed a dropout rate of 0.25 to prevent over-fitting. The output was then passed through a dense layer with a single unit and a sigmoid activation function, producing the probability of pairing between the heavy and light chains, resulting in a binary classification indicating whether the chains were paired (using a threshold of 0.5). The CNN model was built using the Keras library in the TensorFlow framework (13).

Optimisation of CNN model

Optimiser The optimiser is a critical component in the training of the neural networks, to update the weights of the model in order to minimise the loss function. Different optimisers have different strategies to update the weights, and the choice of optimiser can have a significant impact on the performance of the model. Here, we investigated the impact of RMSProp (14) and Adam (15) on the performance of the prediction.

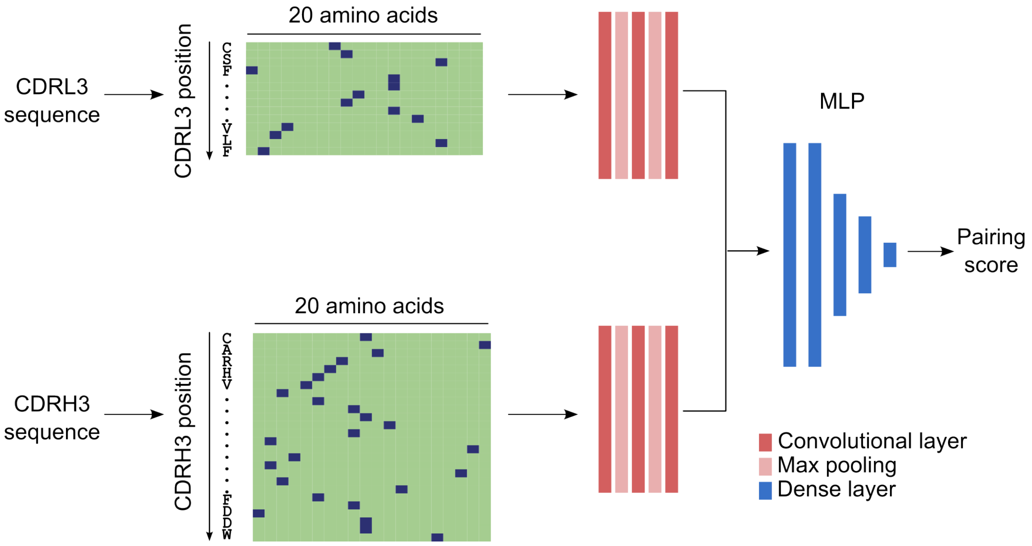

Figure S5: The architecture of the CNN model for pairing prediction.
The CDRH3 and CDRL3 sequences were first one-hot encoded as a matrix of shape `(sequence_length, 20)`, with `1`s being the amino acid at the corresponding position (colored blue in the matrix). Encoded sequence were then fed into a series of convolutional and max pooling layers. The output from the CDRH3 and CDRL3 branches were flattened and concatenated, passing through a set of dense layers (Units: 256, 256, 128, and 64; with ReLu as activation function and a dropout rate of 0.25 for each). The last dense layer consisted of a single neuron with a sigmoid activation function, producing the probability of pairing between the heavy and light chains.

Convolutional layers Increasing the number of convolutional layers is a common strategy to build a deeper network and improve the performance of the CNN model. Here, the performance of the model using 3 and 4 convolutional layers were evaluated.

Residual network Using a deeper neural network by adapting more convolutional layers can improve the performance of the model by allowing the model to learn more complex features. Therefore, we investigated the incorporation of the Residual networks (ResNets) (16) that utilises skip connections between convolutional layers, allowing for a deeper neural network and avoiding the vanishing gradient problem at the same time. Here, the max pooling and the convolutional layer at the end for each CDRH3/CDRL3 branch were replaced by the convolutional and identity block in the ResNet framework (Figure **S6**).

Predicting H-L pairing preference using V region sequences

Language models adopt the transformer architecture, where the attention matrix is utilised to better capture the long-range relationships between the tokens in sentences, or, in the case of proteins or specifically antibodies, residues along the amino acid sequence. Since V region sequences ($\sim$ 100 residues) are longer compared with the CDR3 sequences ($max.$ 26 residues), we investigated the use of language models for the prediction of H-L pairing preference using sequences of the entire VH and VL domains as input. Language models can be used as either as a

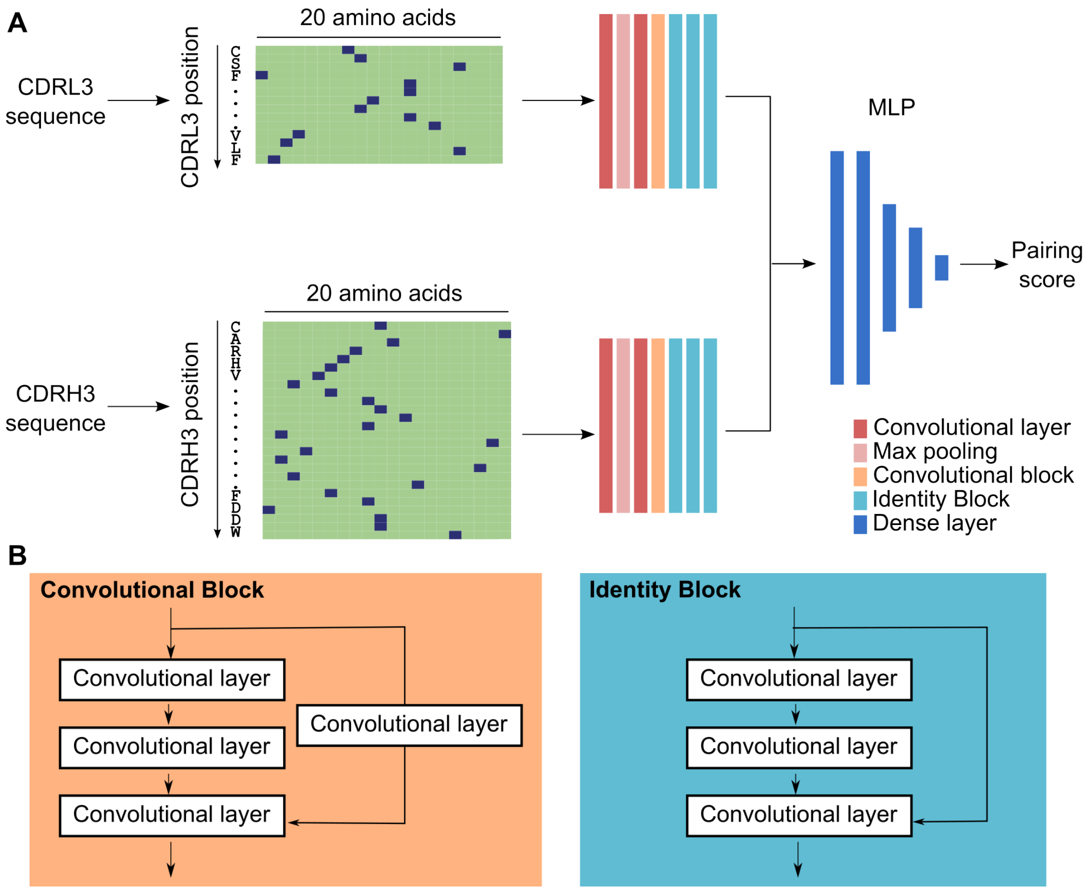

Figure S6: CNN model with ResNet architecture.
(A) The last max pooling and the convolutional layer in the original CNN structure was replaced with one convolutional block and three identity block. **(B)** The convolutional block consisted of three convolutional layers with a kernel size of 3 and 128 filters, with ReLu activation function. The skip function contained a convolutional layer without activation function, and served to transform the dimensions between the input and output of the block. The identity block consisted of three convolutional layers with a kernel size of 3 and 128 filters with ReLu activation function. A Skip function linked between the input and output of the block.

"featuriser", where it encodes the sequence into a fixed-length numeric embedding vector for subsequent machine learning pipelines, or they can also be directly fine-tuned for specific prediction tasks. We investigated both methods in our task, on a generic protein language model (ESM-2 (17)) and an antibody-specific language model (AntiBERTa2 (18)).

Utilising the language model as a featuriser

The VH and VL sequences were separately fed into the language model to output the embedding matrix of shape `(sequence_length, hidden_size)`. Hidden size represents the number of trainable weights for each token, i.e., for each residue. The embedding matrix was then mean-pooled to generate the sentence embedding, where the matrix was averaged along the axis of sequence to produce a fixed-length vector of the shape `(1,hidden_size)`. The two vectors were then concatenated and fed into a multilayer perceptron (MLP) to predict the pairing. The setup for the MLP is the same as in Figure **S5**, with the unit size for the dense layer as 256, 256, 128, 64, and the dropout rate 0.25, respectively. Both ESM-2 (17) and AntiBERTa2 were investigated for this architecture (Table **S4**).

Table S4: Language models used in the investigation for pairing prediction.

Model	Training data	Parameter size	Hidden size
AntiBERTa2	OAS (19) and antibody structures	202.64M	1024
ESM2	UniRef50	150M	640

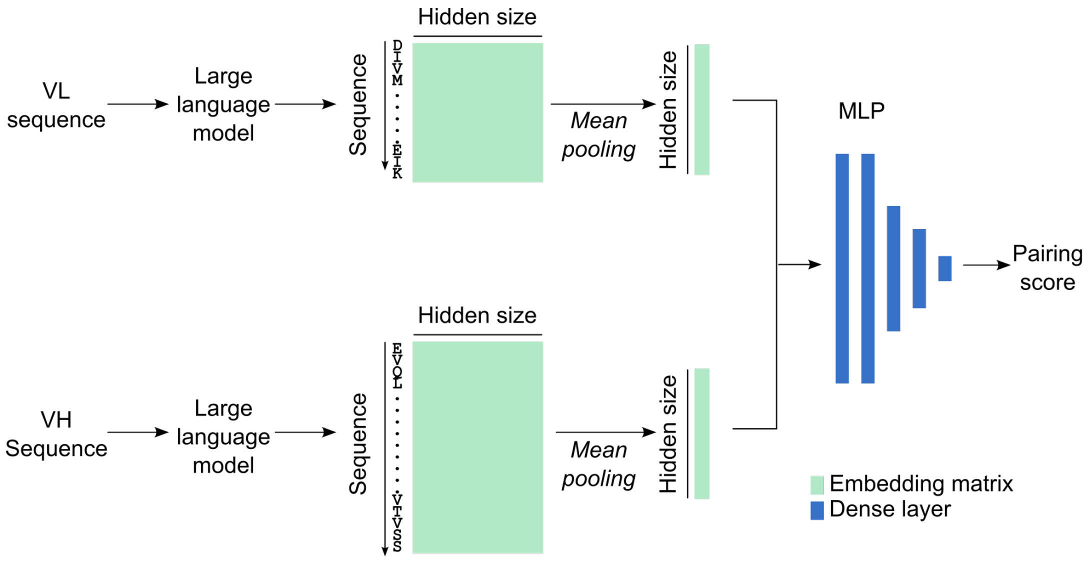

Figure S7: Utilising the language model as a featuriser for pairing prediction.
Paired VH and VL sequences were first embedded by different language models, then the embeddings were mean-pooled to generate the sentence embedding. The two vectors were concatenated and fed into MLP (a series of dense layers) to predict the pairing between the heavy and light chains.

Fine-tuning protein language models for pairing prediction

We also adopted the conventional pipeline to fine-tune ESM-2 and AntiBERTa2 with the training data we collated to predict H-L chain pairing. The VH and VL amino acid sequences were jointly inputted into fine-tuning AntiBERTa2, by adding a separation token (`<SEP>`) between H and L sequences, a typical method used to concatenate sentences in BERT-based model (20); for ESM-2, we concatenated VH and VL sequences by adding three gaps (`-`) in between, as ESM-2 was originally designed for structural prediction (17). The concatenated sequences were then inputted into the model to fine-tune for three epochs with batch size 48 for AntiBERTa2 and batch size 32 for ESM-2 due to the GPU memory limit and computational efficiency.

References

[1] Rajan, S. *et al.* Recombinant human B cell repertoires enable screening for rare, specific, and natively paired antibodies. *Communications Biology* **1**, 1–8 (2018).

[2] DeKosky, B. J. *et al.* Large-scale sequence and structural comparisons of human naive and antigen-experienced antibody repertoires. *Proceedings of the National Academy of Sciences* **113**, E2636–E2645 (2016).

[3] Jaffe, D. B. *et al.* Functional antibodies exhibit light chain coherence. *Nature* **611**, 352–357 (2022).

[4] Phad, G. E. *et al.* Clonal structure, stability and dynamics of human memory B cells and circulating plasmablasts. *Nature Immunology* **23**, 1076–1085 (2022).

[5] James, K. R. *et al.* Distinct microbial and immune niches of the human colon. *Nature Immunology* **21**, 343–353 (2020).

[6] Eccles, J. D. *et al.* T-bet+ Memory B Cells Link to Local Cross-Reactive IgG upon Human Rhinovirus Infection. *Cell Reports* **30**, 351–366.e7 (2020).

[7] Pelanda, R. Dual immunoglobulin light chain B cells: Trojan horses of autoimmunity? *Current Opinion in Immunology* **27**, 53–59 (2014).

[8] Kim, W. *et al.* Germinal centre-driven maturation of B cell response to mRNA vaccination. *Nature* **604**, 141–145 (2022).

[9] Lindeman, I. *et al.* BraCeR: B-cell-receptor reconstruction and clonality inference from single-cell RNA-seq. *Nature Methods* **15**, 563–565 (2018). Publisher: Nature Publishing Group.

[10] Poslavskaya, E. & Korolev, A. Encoding categorical data: Is there yet anything 'hotter' than one-hot encoding? (2023). ArXiv:2312.16930 [cs].

[11] Lefranc, M.-P. *et al.* IMGT unique numbering for immunoglobulin and T cell receptor variable domains and Ig superfamily V-like domains. *Developmental and Comparative Immunology* **27**, 55–77 (2003).

[12] Agarap, A. F. Deep Learning using Rectified Linear Units (ReLU) (2019). ArXiv:1803.08375 [cs].

[13] Martín Abadi *et al.* TensorFlow: Large-scale machine learning on heterogeneous systems (2015). URL https://www.tensorflow.org/.

[14] Graves, A. Generating Sequences With Recurrent Neural Networks (2014). ArXiv:1308.0850 [cs].

[15] Kingma, D. P. & Ba, J. Adam: A Method for Stochastic Optimization (2017). ArXiv:1412.6980 [cs].

[16] He, K., Zhang, X., Ren, S. & Sun, J. Deep Residual Learning for Image Recognition (2015). ArXiv:1512.03385 [cs] version: 1.

[17] Lin, Z. *et al.* Evolutionary-scale prediction of atomic-level protein structure with a language model. *Science* **379**, 1123–1130 (2023).

[18] Barton, J., Galson, J. D. & Leem, J. Enhancing Antibody Language Models with Structural Information (2024).

[19] Olsen, T. H., Boyles, F. & Deane, C. M. Observed Antibody Space: A diverse database of cleaned, annotated, and translated unpaired and paired antibody sequences. *Protein Science* **31**, 141–146 (2022).

[20] Devlin, J., Chang, M.-W., Lee, K. & Toutanova, K. BERT: Pre-training of Deep Bidirectional Transformers for Language Understanding (2019). ArXiv:1810.04805.
