# Supplementary material for: ImmunoMatch learns and predicts cognate pairing of heavy and light immunoglobulin chains
Source: Nat Methods. 2025 Nov 18;23(1):106–17. doi: 10.1038/s41592-025-02913-x (PMC12791012; doi:10.1038/s41592-025-02913-x)
Supplement: Supplementary file 2 — Reporting Summary [file 41592_2025_2913_MOESM2_ESM.pdf]

nature portfolio

Corresponding author(s): Dr. Joseph Ng, Prof. Franca Fraternali

Last updated by author(s): Sep 25, 2025

Reporting Summary

Nature Portfolio wishes to improve the reproducibility of the work that we publish. This form provides structure for consistency and transparency in reporting. For further information on Nature Portfolio policies, see our Editorial Policies and the Editorial Policy Checklist.

Statistics

For all statistical analyses, confirm that the following items are present in the figure legend, table legend, main text, or Methods section.

n/a	Confirmed	
☐	☒	The exact sample size (*n*) for each experimental group/condition, given as a discrete number and unit of measurement
☐	☒	A statement on whether measurements were taken from distinct samples or whether the same sample was measured repeatedly
☐	☒	The statistical test(s) used AND whether they are one- or two-sided<br>*Only common tests should be described solely by name; describe more complex techniques in the Methods section.*
☐	☒	A description of all covariates tested
☐	☒	A description of any assumptions or corrections, such as tests of normality and adjustment for multiple comparisons
☐	☒	A full description of the statistical parameters including central tendency (e.g. means) or other basic estimates (e.g. regression coefficient) AND variation (e.g. standard deviation) or associated estimates of uncertainty (e.g. confidence intervals)
☐	☒	For null hypothesis testing, the test statistic (e.g. F, t, r) with confidence intervals, effect sizes, degrees of freedom and P value noted<br>*Give P values as exact values whenever suitable.*
☒	☐	For Bayesian analysis, information on the choice of priors and Markov chain Monte Carlo settings
☒	☐	For hierarchical and complex designs, identification of the appropriate level for tests and full reporting of outcomes
☒	☐	Estimates of effect sizes (e.g. Cohen's *d*, Pearson's *r*), indicating how they were calculated

Our web collection on statistics for biologists contains articles on many of the points above.

Software and code

Policy information about availability of computer code

Data collection	The manuscript utilised the following paired antibody repertoire sequencing datasets: Rajan et al. (Commun Biol 2018), DeKosky et al. (PNAS 2016), Jaffe et al. (Nature 2022), Phad et al. (Nat Immunol 2022), James et al. (Nat Immunol 2020), Eccles et al. (Cell Rep 2020), King et al. (Sci Immunol 2021), Engblom et al. (Science 2023), Lindeman et al. (Nature Methods 2018), datasets from 10x Genomics (https://www.10xgenomics.com/datasets/human-b-cells-from-a-healthy-donor-1-k-cells-2-standard-6-0-0), Parse Biosciences (https://www.parsebiosciences.com/datasets/bcr-sequencing-of-1-million-healthy-and-diseased-samples-in-a-single-experiment/) and a curated dataset from individual lymphoma and leukaemia samples from the GenBank database. The data were collected and curated manually.
Data analysis	All code to use and apply the ImmunoMatch models, as well as scripts to generate the figures and tables in the manuscript , can be found in the GitHub repository (https://github.com/Fraternalilab/ImmunoMatch). ImmunoMatch is available as a standalone python package (https://pypi.org/project/ImmunoMatch/), as well as interactive notebook on Google Collaboratory (https://colab.research.google.com/github/Fraternalilab/ImmunoMatch/blob/main/Run_ImmunoMatch.ipynb).

For manuscripts utilizing custom algorithms or software that are central to the research but not yet described in published literature, software must be made available to editors and reviewers. We strongly encourage code deposition in a community repository (e.g. GitHub). See the Nature Portfolio guidelines for submitting code & software for further information.

Data

Policy information about availability of data

All manuscripts must include a data availability statement. This statement should provide the following information, where applicable:

- Accession codes, unique identifiers, or web links for publicly available datasets
- A description of any restrictions on data availability
- For clinical datasets or third party data, please ensure that the statement adheres to our policy

Final checkpoints of ImmunoMatch, ImmunoMatch-kappa and ImmunoMatch-lambda are available on HuggingFace at https://huggingface.co/fraternalilab/immunomatch. Code to run ImmunoMatch to annotate sequences can be found on Google Collaboratory (https://colab.research.google.com/github/Fraternalilab/ImmunoMatch/blob/main/Run_ImmunoMatch.ipynb). Data and code to generate figures in this manuscript are available on GitHub (https://github.com/Fraternalilab/ImmunoMatch). A standalone Python package to apply ImmunoMatch is available on PyPI at https://pypi.org/project/ImmunoMatch/.

Research involving human participants, their data, or biological material

Policy information about studies with human participants or human data. See also policy information about sex, gender (identity/presentation), and sexual orientation and race, ethnicity and racism.

Reporting on sex and gender	N/A. No newly generated data is presented in this study. We use publicly available datasets to validate our computational method (see Software and Code)
Reporting on race, ethnicity, or other socially relevant groupings	N/A. No newly generated data is presented in this study. We use publicly available datasets to validate our computational method (see Software and Code)
Population characteristics	N/A. No newly generated data is presented in this study. We use publicly available datasets to validate our computational method (see Software and Code)
Recruitment	N/A. No newly generated data is presented in this study. We use publicly available datasets to validate our computational method (see Software and Code)
Ethics oversight	N/A. We did not generate new data from human samples for this study..

Note that full information on the approval of the study protocol must also be provided in the manuscript.

Field-specific reporting

Please select the one below that is the best fit for your research. If you are not sure, read the appropriate sections before making your selection.

☒ Life sciences ☐ Behavioural & social sciences ☐ Ecological, evolutionary & environmental sciences

For a reference copy of the document with all sections, see nature.com/documents/nr-reporting-summary-flat.pdf

Life sciences study design

All studies must disclose on these points even when the disclosure is negative.

Sample size	We use all sequences available to us from high-quality paired antibody repertoire datasets. We clustered sequences prior to defining training and test datasets to avoid skewing of model learning towards large expanded B cell clonotypes.
Data exclusions	N/A. All collected datasets were used in model training and validation following standard machine learning practices to ensure model generalisability on external datasets.
Replication	Whenever possible k-fold cross validation procedure was employed to demonstrate model consistency on different data subsets. The prediction model was validated on separate datasets representing a variety of biological contexts.
Randomization	Sequences were randomly divided into training and test sets following standard machine learning practices. We clustered CDR sequences using the CD-HIT (Fu et al. Bioinformatics 2012) algorithm prior to defining the training and test sets to exclude the chance of similar sequences appearing in both datasets and therefore minimise data leakage. These details are included in the Methods section. Randomisation in the validation datasets is not applicable.
Blinding	Blinding is not relevant to this study since our main aim is not to claim differences between defined groups of observations.

Reporting for specific materials, systems and methods

We require information from authors about some types of materials, experimental systems and methods used in many studies. Here, indicate whether each material, system or method listed is relevant to your study. If you are not sure if a list item applies to your research, read the appropriate section before selecting a response.

Materials & experimental systems

n/a	Involved in the study
☒	☐ Antibodies
☒	☐ Eukaryotic cell lines
☒	☐ Palaeontology and archaeology
☒	☐ Animals and other organisms
☒	☐ Clinical data
☒	☐ Dual use research of concern
☒	☐ Plants

Methods

n/a	Involved in the study
☒	☐ ChIP-seq
☒	☐ Flow cytometry
☒	☐ MRI-based neuroimaging

Plants

Seed stocks	N/A
Novel plant genotypes	N/A
Authentication	N/A
